# Evidence for Glutamate as a Neuroglial Transmitter within Sensory Ganglia

**DOI:** 10.1371/journal.pone.0068312

**Published:** 2013-07-02

**Authors:** Ling-Hsuan Kung, Kerui Gong, Mary Adedoyin, Johnson Ng, Aditi Bhargava, Peter T. Ohara, Luc Jasmin

**Affiliations:** 1 Department of Anatomy, University of California San Francisco, San Francisco, California, United States of America; 2 School of Biomedical Sciences, Faculty of Medicine, The Chinese University of Hong Kong, Hong Kong, China; 3 Department Surgery, University of California San Francisco, San Francisco, California, United States of America; The Hebrew University Medical School, Israel

## Abstract

This study examines key elements of glutamatergic transmission within sensory ganglia of the rat. We show that the soma of primary sensory neurons release glutamate when depolarized. Using acute dissociated mixed neuronal/glia cultures of dorsal root ganglia (DRG) or trigeminal ganglia and a colorimetric assay, we show that when glutamate uptake by satellite glial cells (SGCs) is inhibited, KCl stimulation leads to simultaneous increase of glutamate in the culture medium. With calcium imaging we see that the soma of primary sensory neurons and SGCs respond to AMPA, NMDA, kainate and mGluR agonists, and selective antagonists block this response. Using whole cell patch-clamp technique, inward currents were recorded from small diameter (<30 µm) DRG neurons from intact DRGs (*ex-vivo* whole ganglion preparation) in response to local application of the above glutamate receptor agonists. Following a chronic constriction injury (CCI) of either the inferior orbital nerve or the sciatic nerve, glutamate expression increases in the trigeminal ganglia and DRG respectively. This increase occurs in neurons of all diameters and is present in the somata of neurons with injured axons as well as in somata of neighboring uninjured neurons. These data provides additional evidence that glutamate can be released within the sensory ganglion, and that the somata of primary sensory neurons as well as SGCs express functional glutamate receptors at their surface. These findings, together with our previous gene knockdown data, suggest that glutamatergic transmission within the ganglion could impact nociceptive threshold.

## Introduction

Glutamate is the common excitatory neurotransmitter of the central and peripheral nervous systems and is found in both nociceptive as well as non-nociceptive sensory pathways [1], [2], [3], [4]. The ubiquitous distribution of glutamate has made it difficult to formulate strategies that could target glutamatergic transmission involved in a specific function such as nociception while leaving other processes intact. Thus the possibility of targeting glutamatergic transmission in the peripheral nervous system has been suggested given that small diameter primary sensory neurons, many of which are nociceptive, express glutamate and glutamate receptors [5], [6], [7]. Activation of these neurons leads to glutamate release at their central as well as peripheral terminals [8], [9], [10], and nociception [11], [12].

The possibility that glutamate is also released within the sensory ganglion is contentious as there are no synapses on the soma of primary sensory neurons. Clearly, the cell membranes of primary sensory neuron somas contain ionotropic (iGluR) and metabotropic (mGluR) receptors [13], [14], [15], [16]. Moreover, the machinery for production, release, and recycling of glutamate is present in sensory ganglia including the amidohydrolase enzyme, glutaminase [17], [18], vesicular glutamate transporters (VGLUT1, 2 and 3) [19], [20], the glutamate aspartate transporter (GLAST) and glutamate transporter 1 (GLT1) [21], as well as the recycling enzyme glutamine synthetase [17], [22]. This, and the presence of glutamate within the soma would allow for local non-synaptic glutamatergic transmission. We have indirect evidence for non-synaptic transmission from experiments in the trigeminal ganglion in which a glutamate-glutamine cycle enzyme or a glutamate uptake transporter were knocked-down using double stranded RNA [22], [23]. These studies showed that the knockdown was confined to the local satellite glial cells (SGCs) and that pain behavior was consistently altered, which can be best explained by a change in intraganglionic glutamatergic transmission.

The goal of the present study was to determine if glutamate is released by the soma of primary sensory neurons and if functional glutamate receptors are present at the surface of the soma of these neurons. It has generally assumed that glutamate vesicles and receptor proteins found in the soma of primary sensory neurons are destined for transport to axon terminals and that functional glutamate receptors are not inserted onto the somatic membrane. Evidence from *in vitro* studies, however, suggests that the soma of primary sensory neurons can release glutamate [24], [25], [26] and express functional NMDA receptors at the surface [27], supporting the presence of intraganglionic glutamatergic transmission [1]. There is precedence for non-synaptic release of other neurotransmitters and neuromodulators within sensory ganglia. Examples are adenosine triphosphate (ATP), possibly calcitonin gene related peptide and tumor necrosis factor-alpha [28], [29], [30]. These substances may be released by neurons or glia and their action would occur locally on one or both types of cells.

It is not known whether or not glutamate receptors other than the NMDA receptors are present at the somatic membrane of primary sensory neurons or whether glutamate receptors are also present on SGCs. To further examine these questions we used a combination of calcium imaging and glutamate measurements on acute mixed cultures of adult sensory ganglion together with patch clamp recordings on intact whole sensory ganglia *ex-vivo*. Our results reveal that multiple functional glutamate receptors are present at the somatic surface of both neurons and SGCs in the ganglion. We confirmed that primary sensory neuron somas could release glutamate when they are acutely depolarized. An unexpected finding was that the glutamate immunoreactivity increases in the soma of primary sensory neurons in sensory ganglia after chronic constriction nerve injury (CCI).

## Methods

### Animals

Adult male Sprague-Dawley rats were used. For CCI-surgery the rats weighed 270–290 grams and for DRG culture 150–200 grams. All animals were housed on a 12-hour light–dark cycle and given food and water *ad libitum*.

### Ethics

Procedures for the maintenance and use of the experimental animals conformed to the regulations of UCSF Committees on Animal Research and were carried out in accordance with the guidelines of the NIH regulations on animal use and care (Publication 85-23, Revised 1996). The UCSF Institutional Animal Care and Use Committee approved the protocols used in this study.

### Surgery

#### Chronic Constriction Injury (CCI) of the INFRA-orbital NERVE (ION)

The procedure for CCI of the ION has been described in detail previously [31]. Briefly, rats were anesthetized with a mixture of 90 mg/kg ketamine (Fort Dodge Animal Health, Overland Park, KS, USA) and 10 mg/kg xylazine (Akorn Inc., Gurnee, IL, USA) and placed in a stereotaxic head holder. Forty percent oxygen was delivered through a facemask. The skull and nasal bone were exposed through a 2 cm supra-orbital skin incision. The superficial muscle was detached from the superior edge of the orbit. Gentle retraction of the orbital contents exposed the ION. Two 5-0 chromic gut ligatures were loosely tied 2 mm apart around the exposed nerve using the operating microscope and microsurgical techniques. For sham-operated animals, the ION was exposed but no ligatures were placed. The skin incision was closed using interrupted 6-0 silk sutures.

#### Chronic Constriction Injury (CCI) of the Sciatic Nerve (SN)

We followed the procedure as originally described by Bennett [32]. Surgery was carried out on rats placed under anesthesia using 2% isoflurane (Solvay, Mendota Height, MN, USA) and 40% oxygen delivered through a facemask. The middle third of the left sciatic nerve was exposed through a 1.5 cm longitudinal skin incision. Four 4-0 chromic gut ligatures (Ethicon, Somerville, NJ, USA) were loosely tied around the sciatic nerve using the operating microscope and microsurgical techniques. The skin was then closed with interrupted 3-0 silk sutures.

### Tissue Processing

Histology. Rats were perfused at 4, 7, and 14 days after CCI-ION and at 7 and 21 days after CCI-SN under pentobarbital anesthesia (100 mg/kg i.p.). Perfusions were performed transcardially with 400 mL Tyrode’s solution followed by 400 mL of 4% paraformaldehyde in phosphate buffer at pH 7.4. Both the ipsilateral and contralateral trigeminal ganglia or lumbar dorsal root ganglia, along with samples of brain and spinal cord, were removed and post-fixed in 4% paraformaldehyde for 3–5 hours, and then cryo-protected with 30% sucrose in PBS (pH = 7.4) for at least 48 hours. Ganglia from different rats were embedded in the same block and cut together to minimize processing variation for quantification. Ten microns (10 µm) longitudinal sections of ganglia were cut with a cryostat.

#### Immunohistochemistry

Optimal dilutions and incubation times for all primary and secondary antibodies were determined prior to use and control sections with the primary antibody omitted were used to test for antibody specificity. All slides to be compared for quantification were processed at the same time. The following primary antibodies were used at the corresponding dilutions: rabbit anti-glutamate 1∶8000 (#1766 Arnel Products Co., Inc, NY, USA); mouse anti-beta III tubulin, 1∶20,000 (#G7121, Promega, Madison, WI, USA); rabbit anti-activating transcription factor 3 (ATF3), 1∶800 (#sc-188, Santa Cruz, Dallas, TX, USA); rabbit anti-glutamine synthetase 1∶25000 (#G2781, Sigma, St. Louis, MO, USA); mouse anti-glutamine synthetase, 1∶1000 (#MAB302, Chemicon/Millipore, Billerica, MA, USA); rabbit anti-cellubrevin 1∶500, (#104103, Synaptic Systems, Goettingen, Germany); rabbit anti-SNAP25, 1∶500 (#111002 Synaptic system, Goettingen, Germany); guinea pig anti-synaptobrevin-2 1:200 (#104204, Synaptic Systems, Goettingen, Germany); rabbit anti-SCAMP1 1:400 (#121002, Synaptic Systems, Goettingen, Germany); rabbit anti-NMDA receptor-1 (NR2A) 1∶1000 (AB9864, Millipore, Billerica, MA, USA); rabbit anti-GluR6 (GluK2) 1∶5000 (ab124702, Abcam, Cambridge, MA); rabbit anti-GluR4 (GluA4) 1∶500 (#AB1508, Millipore, Billerica, MA, USA); guinea pig anti-mGluR8 1:2000 (#AB5362, Chemicon/Millipore, Billerica, MA, USA). The tissue was incubated in primary antibodies at room temperature overnight, then washed 3 4 times before being incubated for 30 minutes in a humidified chamber with species-specific secondary antibodies conjugated to FITC or CY3 and diluted 1∶500. The slides were then washed and cover-slipped with Vector shield mounting medium plus DAPI. Slides were analyzed on both a conventional fluorescence microscope and a confocal microscope.

### Behavioral Testing

CCI of ION; von Frey Hair testing. Three von Frey hairs of increasing stiffness, 2, 10, and 50 g were used. Five or six consecutive applications were performed at 5 s intervals on different areas of the vibrissal pad and in the perioral and perinasal territory. The observer scored the behavioral response of the rats based on the method of Vos et al. [33] as follows: 0, no detection; 1, detection and exploration of the von Frey hair (sniffing, licking); 2, head withdrawal and/or single grabbing movement; 3, attack (includes biting) and/or escape (includes burrowing) and/or multiple grabbing movements; and 4, active asymmetrical grooming (at least three face wash strokes) directed toward the stimulated facial area. For each hair, the highest score was recorded and the results are presented as the average of the scores obtained from the three different hairs. The average value is presented because the analysis of scores from each hair separately gave the same results when compared between groups.

#### CCI of the sciatic nerve SN; von Frey Hair testing

Testing was done with a von Frey hair algometer [34]. Briefly, individual rats were placed in a Plexiglas enclosure (30×35×13.5 cm) equipped with a metal grid floor with openings of 0.6×0.6 cm. A hand-held transducer with a 1.0-mm nylon probe was gently applied through the metal grid to the plantar aspect of each hindpaw and progressively pushed vertically until a paw withdrawal occurred or cut-off was reached (100 g). The applied force was displayed on a meter attached to the transducer (IITC, Inc., model 1601, www.iitcinc.com).

### Cell Culture

Male Sprague-Dawley rats (150–200 grams) were anaesthetized with pentobarbital (40 mg/kg, i.p.) and lumbar levels of spinal cord dorsal root ganglia were dissected and placed into DMEM/10% FBS containing penicillin (100 U/ml) and streptomycin (100 µg/ml), followed by three-hour incubation at 37°C and 5% CO_2_ with 0.125% collagenase B (Roche, Indianapolis, IN, USA), then 30 minutes additional incubation with 0.25% trypsin (Sigma, St. Louis, MO, USA). Afterward DNase I (90 µg/mL; Sigma, St. Louis, MO, USA) and soybean trypsin inhibitor (100 µg/mL; Sigma, St. Louis, MO, USA) were added, and the cells dispersed by trituration using a flame-polished salinized (4% dimethyl-dichlorosilane in toluene) Pasteur pipette. The resulting cell suspension was centrifuged 1200 rpm (96×g, 20°C) through a cushion of 15% bovine serum albumin (BSA) (Sigma, St. Louis, MO, USA) in order to eliminate most of the cellular debris. The cell pellet was re-suspended in DMEM and centrifuged to remove the BSA. Using a hemocytometer to determine neurons density, we seeded 5000 neurons per well onto glass 12 mm cover slips pre-coated with poly-DL-ornithine (500 µg/mL; Sigma, St. Louis, MO, USA) and laminin (5 µg/mL; Sigma, St. Louis, MO, USA) in 24-well tissue culture plates at 37°C and 5% CO_2_. Cells were fixed after 24 hours for immunocytochemistry staining. For the Ca^2+^ imaging experiments, mixed cultures were plated on 35 mm diameter coverslips in 6-well culture plates and used for experiments within 24 to 48 hours.

### Glutamate Measurement

Glutamate levels in culture were detected using an enzymatic assay similar to that described by Akagi and colleagues [35]. In the presence of glutamate, glutamate oxidase reduces NAD^+^ to NADH and produces α-ketoglutarate, NH_4_^+^, and H_2_O_2_. The Amplex® UltraRed reagent (Invitrogen, Grand Island, NY, USA) reacts with H_2_O_2_ in the presence of horseradish peroxidase to produce bright fluorescence with long-wavelength spectra (Ex. 568 nm, Em. 581 nm) [35] that can be measured in a spectrofluorometer.

DRG mixed cultures described above were seeded in 96 well plates at a density of 30,000 neurons per well. Due to the high density of neurons in each well, the culture medium was changed every 6–8 hours for 3 days. To avoid glia over population, we used neurobasal and SM2 as the feeding medium to maintain healthy neurons and prevent overgrowth of the SGC population. Three different treatments were included in this study. Group 1 had no KCl stimulation to serve as a control. Group 2 received 100 µM KCl to activate neurons. Group 3 was pretreated with DL-*threo*-β-Benzyloxyaspartic acid (TBOA 100 µM, Tocris, Minneapolis, MN, USA) for 2 hours, which blocks glutamate uptake, followed by KCl stimulation. All reaction agents, excluding TBOA, were diluted in HEPES medium (mM: 129 NaCl, 5 KCl, 2 CaCl_2_, 1 MgCl_2_, 30 glucose, 25 HEPES) and pH-adjusted to 7.4 with NaOH. TBOA was incubated with culture medium for 2 hours in group 3 cells prior to KCl stimulation.

Procedure: After 72 hours culture, the medium in all three groups was replaced with fresh incubation media containing glutamate oxidase (0.05 U/mL; Sigma, St. Louis, MO) and NAD^+^ (1 mM; Sigma, St. Louis, MO, USA) with or without KCl (100 µM). Standard solutions of HEPES containing glutamate (0–80 µM) containing glutamate oxidase and NAD^+^ were used to determine a concentration curve. Two minutes later, the incubation medium was collected from each well and 1 U/ml horseradish peroxidase (Sigma, St. Louis, MO, USA) and 50 µM Amplex® UltraRed (Molecular Probes Inc., Eugene, OR, USA) were added to each sample. Fluorescence intensity was measured by spectrofluorometry (FlexStation 3, Molecular Devices, LLC, Sunnyvale, CA, USA). Protein concentrations were measured with Pierce BCA Protein Assay Kit (Thermo Scientific, Rockford, IL, USA).

One-way ANOVA was used to determine if there was significance between groups and Newman-Keuls post hoc test were carried out. Each column represents the mean ± S.E.M. of total 6 wells/condition, two different cultures from two different animals.

### Calcium Imaging

All drugs used in the *in vitro* experiments, unless indicated otherwise, were purchased from Tocris (Tocris, Minneapolis, MN, USA). DRG mixed cultures (obtained as above) were loaded with 1 µM of Ca^2+^indicator Fura-2AM and 1 µM of Pluronic F-127 (Life Technologies, Carlsbad, CA, USA) and incubated at 37°C for one hour. After washing off excess dye, cells were allowed to complete de-esterfication for at least 30 minutes at 37°C. DRG mixed cultures were then placed in a recording chamber (POCmini Chamber system, PeCon, Erbach, Germany) and continuously perfused with normal bath solution (mM: 129 NaCl, 5 KCl, 2 CaCl_2_, 1 MgCl_2_, 30 glucose, 25 HEPES) and pH-adjusted to 7.4 with NaOH [36] using a perfusion system (AutoMate Scientific, Berkeley, CA, USA). Fluorescence data were acquired using Piper control software via a CCD camera at 3 frames/second (XR/MEGA-10, Stanford Photonics Inc., Palo Alto, CA, USA). The level of intracellular Ca^2+^ ([Ca^2+^]_i_) elicited by stimuli were measured by the ratio of the emission (510 nm) from Fura-2 excitation at 340 nm and 380 nm. The [Ca^2+^]_i_ concentration induced by ionomycin at the end of each experiment was taken as the maximum 340/380 ratio and set as 1.

All drugs to be tested were applied directly using a hand-held Eppendorf pipette. The chamber contained 1 ml of HEPES to which the drug was added diluted in 500 µl of HEPES to giving the target concentration indicated in Table 1. Prior to drug application, 500 µl of HEPES buffer was infused to control for mechanical calcium transients. The target concentrations in the chamber for all agonists were: Glutamate (200 µM), AMPA (50 µM), NMDA (100 µM), DHPG (mGluR group I agonist, 100 µM), and kainic acid (KA) (30 µM). The concentrations of the respective antagonists were: CNQX (100 µM), APV (100 µM), AP3 (1 mM) and UBP310 (0.5 µM). Cells were recorded for baseline HEPES medium (prior to drug application) for at least 5 minutes, then the agonist applied and they were recorded for 10 minutes. After washing for 10 minutes, the antagonists CNQX, APV, AP3, UBP310 were then individually applied for 10 minutes prior to a second application of the appropriate agonist. Next, following a 10-minute wash, KCl (50 mM) was perfused for 2 minutes to identify the neuronal population. Lastly, ionomycin (20 µM) was added to the medium to verify the viability of cells. Cells that did not respond to ionomycin were excluded from the experiment.

**Table 1:** Agonists and antagonists used in calcium image and patch clamp experiments.

Agonist	Calcium Image	*Ex-vivo* Patch Clamp	Antagonist	Calcium Image	*Ex-vivo* Patch Clamp
Glutamate	200 µM	1 mM	APV+CNQX	-	50 µM+10 µM
NMDA	100 µM	100 µM	APV	100 µM	50 µM
AMPA	50 µM	100 µM	CNQX	100 µM	10 µM
KA	30 µM	100 µM	UBP310	0.5 µM	0.5 µM
DHPG	100 µM	1 mM	AP3	1 mM	60 µM

KA (kainic acid, kainate receptor agonist), DHPG (mGluR1/5 agonist), APV (NMDA antagonist), CNQX (AMPA/Kainate receptor antagonist), AP3 (mGluR1/5 antagonist), UBP310 (Kainate receptor specific antagonist).

Baseline value were acquired by averaging the 340/380 ratio 10 seconds prior to drug application and compared to the peak value which is 10 seconds of the maximum peaks response following drug application. The equation to get the % increase response from baseline is: (peak value – baseline value)/baseline value x100. The ratio of 340/380 responses to drugs between neurons and glia was compared before and after glutamate was added using paired Student’s *t*- test. The same test was also used to determine the difference between agonist effect alone and agonist effect in the presence of the antagonist for the same cell.

### Intact Dorsal Root Ganglion (ex-vivo) Preparation and Whole Cell Patch Clamp Recording

For collecting intact DRGs, rats were first deeply anaesthetized with pentobarbital (40 mg/kg, i.p.). The L4 and L5 DRG were removed and placed into artificial cerebral spinal fluid (aCSF). The aCSF contained (in mM): 124 NaCl, 2.5 KCl, 1.2 NaH_2_PO_4_, 1.0 MgCl_2_, 2.0 CaCl_2_, 25 NaHCO_3_ and 10 glucose in sterile preservative free deionized water. The connective tissue surrounding the DRG was carefully removed under high magnification, and then the ganglia were incubated with a mixture of 0.4 mg/mL trypsin and 1.0 mg/mL type-A collagenase (Sigma, St. Louis, MO USA) for 40 minutes at 37°C to remove the remaining epineurium. The ganglia were then incubated in aCSF bubbled with 95% O_2_ and 5% CO_2_ at room temperature for at least 1 hour before being transferred to the recording chamber. Neurons were visualized with a 40X water-immersion objective using a microscope (FN-600, Nikon, Japan) equipped with infrared differential interference contrast optics. The image was detected with an infrared-sensitive CCD (IR-1000, Dage MTI, USA) and displayed on a monochrome video monitor. Whole-cell current recordings were acquired with an Axon200B amplifier (Molecular Devices, Sunnyvale, CA, USA). Patch pipettes were pulled from borosilicate glass capillaries (BF150-86-10, Sutter Instruments) on a puller (P97, Sutter Instruments, Novato, CA, USA). The resistance of the pipette was 4–5 MΩ when filled with the pipette solution which contained (in mM): 140 KCl, 2 MgCl_2_, 10 HEPES, 2 Mg-ATP, 0.5 Na_2_GTP, pH = 7.4. Osmolality was adjusted to 290–300 mOsm.

After a giga seal was established, the membrane was broken and neurons with a resting membrane potential below −50 mV were selected for further study. The access resistance was 10–20 MΩ and continuously monitored and data were discarded if the access resistance changed more than 15% during an experiment. For all agonists induced currents, the neurons were clamped at −70 mV except for NMDA neurons, which were clamped at −40 mV. Data were acquired with a Digidata 1440 A acquisition system (Molecular Devices) and pCLAMP 10.2 software (Molecular Devices). Signals were low-pass filtered at 5 kHz, sampled at 10 kHz, and analyzed offline.

#### Drug application

All drugs used in the *ex-vivo* experiments were purchased from Tocris (Tocris, Minneapolis, MN, USA). Drugs were dissolved in ultra-pure deionized water as stock solutions and refrigerated for later use. All stock solutions were diluted with aCSF before use.

Table 1 shows the target concentration of glutamate (1 mM), AMPA (100 µM), NMDA (100 µM), KA (100 µM) and DHPG (1 mM) were applied with focal pressure ejection via a puffer pipette controlled by a Picospitzer II (200 ms puff at 1–2 psi; General Valve Inc) to activate the receptors. The corresponding receptor antagonists (Table 1), APV (50 µM)+CNQX (10 µM ), APV (50 µM), CNQX (10 µM), AP3 (60 µM) and UBP310 (0.5 µM) were bath-applied for at least 5 minutes to test the blocking effect. In order to test the blocking effect of receptor antagonists, the responses induced by each agonist before antagonist application were set as 100%, and the currents after antagonist application were expressed as the percentage of previous response. All results were presented as the mean ± SEM. The paired Student’s *t*- test was used to compare agonist induced inward currents and antagonists induced inward currents. Tau of decay is calculated as the time from 100% of peak value to 50% of peak value.

### Quantification of Glutamate Expression

Quantification was done on images taken at the same magnification from glass slides that contained sections of the sensory ganglia ipsilateral and contralateral to the CCI. All images were acquired using the same microscope and camera setting. The Image J (version 1.44, rsbweb.NIH.gov/IJ/) software was used to determine a threshold (see Results) for which glutamate staining was considered to be above background. The same threshold was then used for all ganglia on the same slide that included both ipsilateral and contralateral ganglia from all time points. For each slide a new threshold was set in order to normalize for variation in tissue processing. The total number of pixels was determined for each section to quantify the glutamate staining (i.e. above threshold). For measuring changes in glutamate immunolabeling related to cell size, the area was calculated from the measured diameter of those cells above background in which the nucleus was visible.

For quantification on the trigeminal ganglia we used 3 sections per rat (2 sham and 3 CCI-ION animals per group). For DRG glutamate expression quantification we used 3 sections at each spinal level from 3 animals at 7 and 21 days post CCI. Cells were characterized as small if their cross-sectional surface area was less than 800 µm^2^, medium if the area was 800–1800 µm^2^, and large if the area greater than 1800 µm^2^ [37]. Percent changes in immunoreactive pixel area from paired ipsi/contralateral sides for each sensory ganglion were compared using individual Student’s *t*- test for each time point (4, 7 or 14 days).

## Results

### Glutamate is Released from Mixed DRG Cell Culture

**Figure 1:**
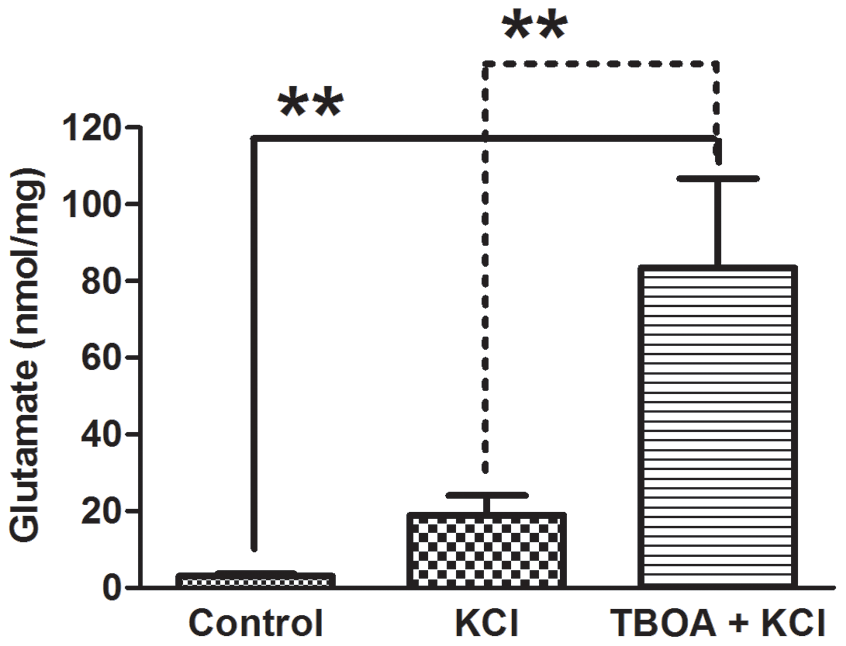
Glutamate assay of dissociated DRG cultures. Colorimetric assay shows that following KCl (100 µM) application to the culture medium there was an increase in glutamate release which did not reach significance compared to control (no stimulus/HEPES only). Following pretreatment with a glutamate transporter blocker (TBOA 100 µM), KCl stimulation resulted in a significant increase in extracellular glutamate concentration. Each column represents the mean of 3 wells per condition. Each column represents the mean ± S.E.M. One-way ANOVA **, P<0.01.

In this experiment, we asked two questions: whether neuronal stimulation can induce glutamate release from somas and whether blocking glutamate uptake will increase the extracellular concentration of glutamate. We used a colorimetric assay to detect glutamate released into the medium of a mixed neuronal/SGC culture. Data were collected from six wells per treatment and cultures from 3 different animals. Following 2 minutes of stimulation by addition of 100 mM KCl, the extracellular glutamate concentration increased (Figure 1; 18.9 nmol/mg ±5.2 vs. 3.1 nmol/mg ±0.6) but was not significantly different from control. Because glial cells in the mixed culture rapidly take up extracellular glutamate, we then pretreated cultures with TBOA in order to block glial glutamate uptake. Addition of 100 mM KCl for 2 minutes to these cultures resulted in a significant elevation of extracellular glutamate (83.4 nmol/mg ±23.4) compared to buffer control (3.1 nmol/mg ±0.6) or KCl stimulation without TBOA (18.9 nmol/mg ±5.2) (P<0.001) conditions.

### Neurons and SGCs are Activated by Glutamate

**Figure 2:**
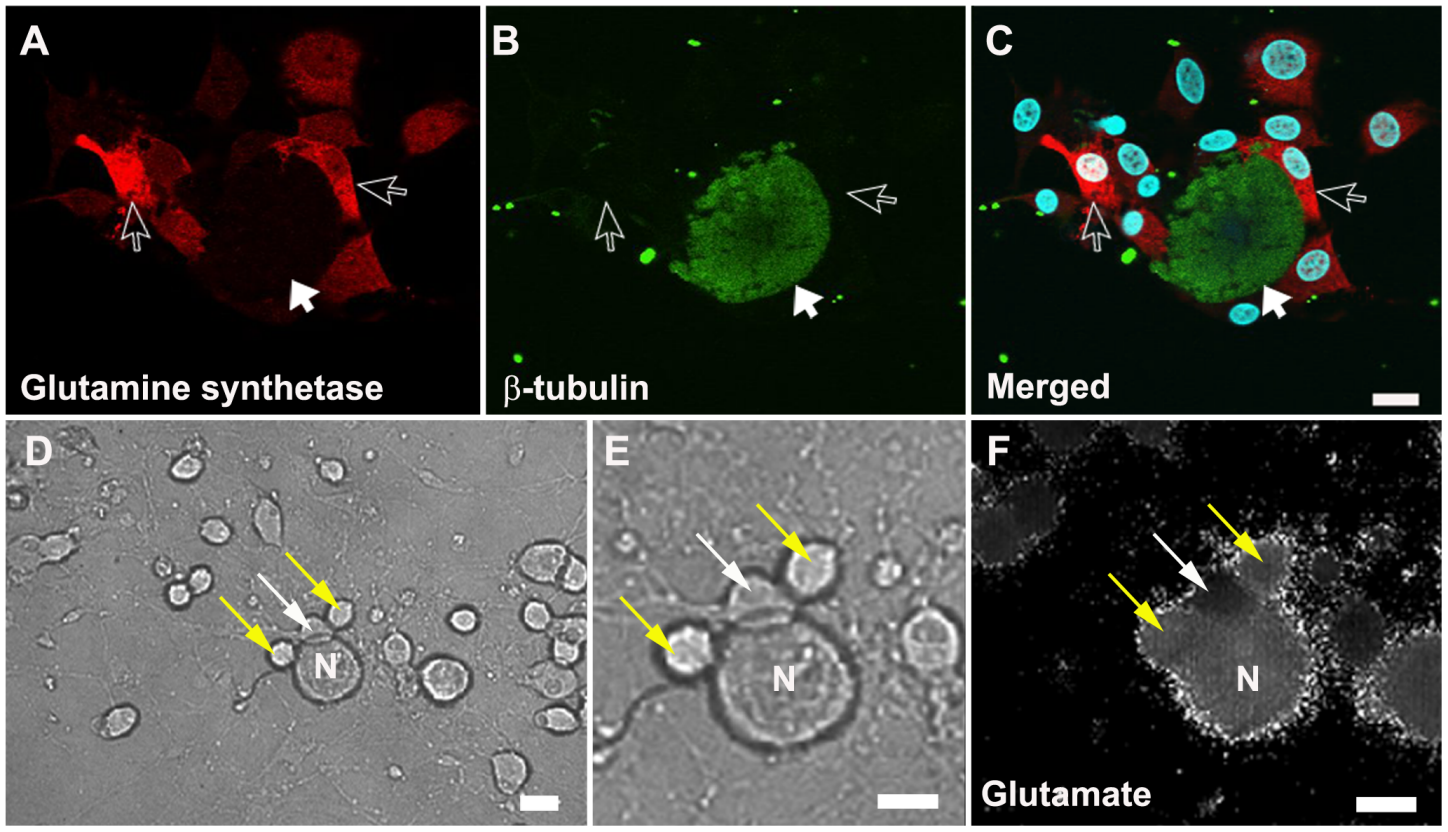
Confocal and calcium influx images of a 24-hour DRG primary mixed culture. A–C confocal image showing SGCs (red: glutamine synthetase an SGC marker, open arrow), neuron (green: β-tubulin, solid arrow) and nucleus (blue: DAPI). D–F representative pictures from a 24-hour culture used for calcium imaging. D and E (high magnification) were taken before the start of calcium imaging. F, fluorescent image following 200 µM glutamate application showing an increase in the 340/380 ratio indicated by an increase in signal intensity in the neuron (N), and two SGCs (yellow arrows) compared to the SGC which did not respond (white arrow). C, D, E, F, scale bar: 10 µm.

**Figure 3:**
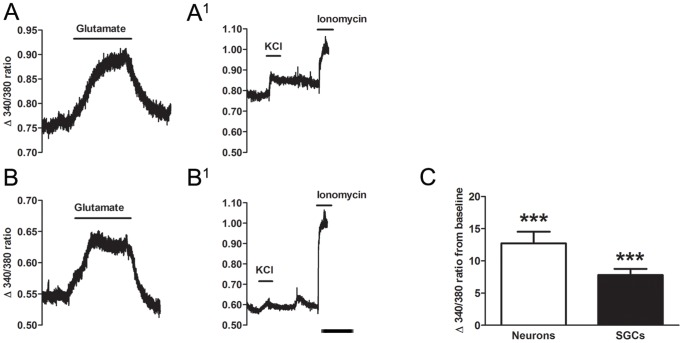
Representative calcium imaging fluorescent traces of a neuron and SGC. A, B, following application of 200 µM glutamate both neurons and SGCs showed an increased fluorescent ratio during the 10 minutes of recording. A^1^, B^1^ after washing with HEPES buffer, KCl (50 mM) was given to identify neurons in the recorded field. Ionomycin (20 µM) was added to test for cell viability at the end of the experiment. Lines show the duration of application for experimental agent. Scale bar = 5 minutes. C, average relative glutamate induced maximum change of 340/380 fluorescence ratio from pre-drug (baseline, normalized at 0) condition. Paired Student’s *t*- tests were used to compare pre-drug and post-drug conditions. Mean ± S.E.M. ***P<0.001.

To examine whether neurons and glial cells in the DRG express functional glutamate receptors, we measured glutamate evoked cytoplasmic Ca^2+^ responses in 24 hr dissociated mixed (neurons and SGCs) DRG cultures (Figure 2). Neurons were identified by their diameter (large >30 µm, small <30 µm) and by their immediate Ca^2+^ influx in response to KCl (50 mM) application. SGCs were recognized by characteristic close association with neurons and by their much smaller diameter (i.e. <10 µm). Figure 3 shows representative traces of neurons (A) and SGCs (B) following addition of glutamate agonists to the culture. Glutamate (200 µM) applied to 1 day mixed primary dorsal root ganglion culture induced an increase in cytosolic Ca^2+^ of both large and small neurons 12.7±1.8 (Figure 3A, C) and SGCs 7.81±1 (Figure 3B, C). Fifty-one out 56 (91%) neurons and 34 out of 40 (85%) SGCs responded to glutamate application. This effect was not due to the mechanical effect of applying the drug as the 340/380 ratio did not change after direct application of HEPES buffer alone (data not shown).

### DRG Perikarya and SGCs have Functional Ionotropic and Group I Metabotropic Receptors

**Figure 4:**
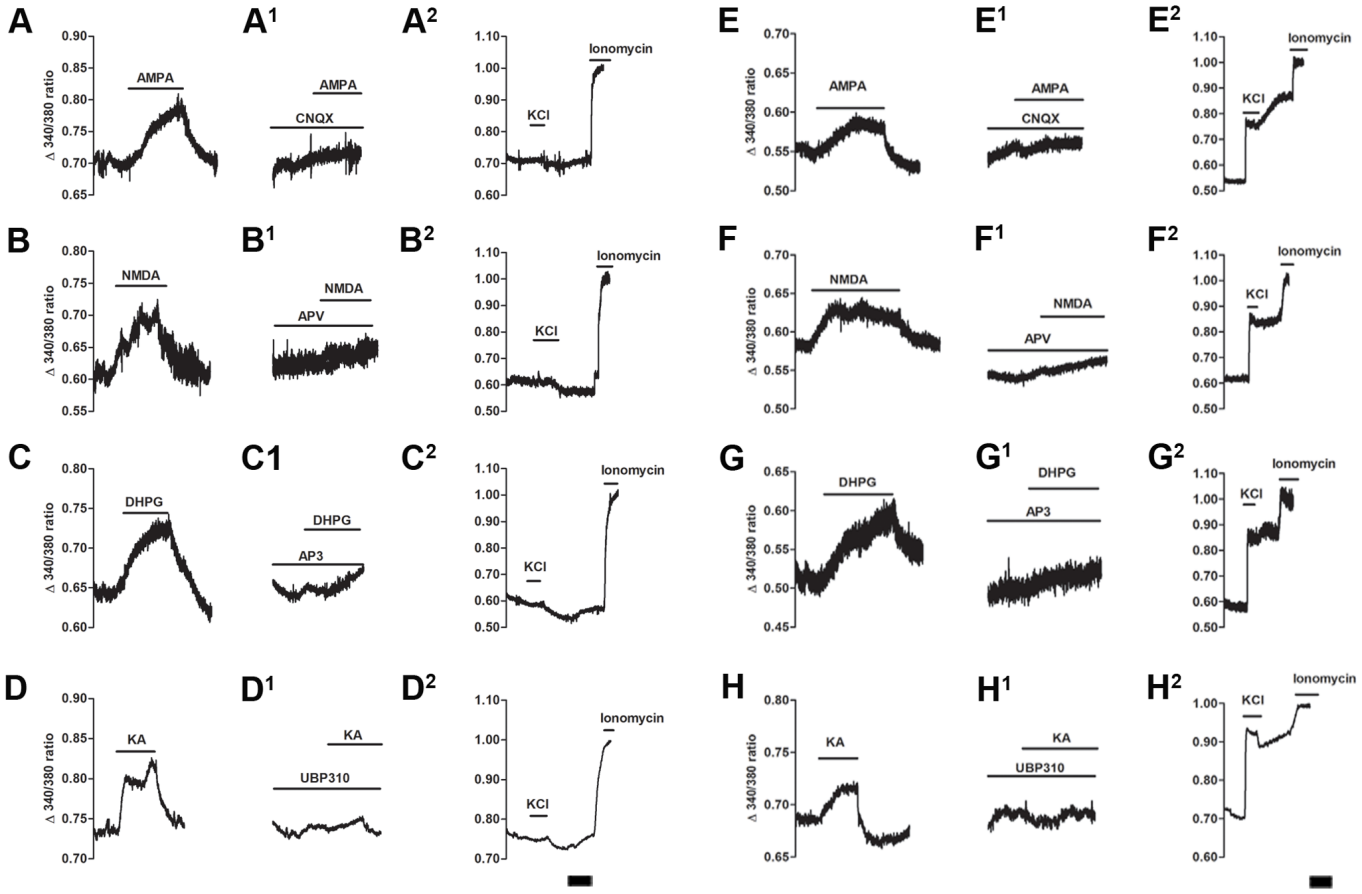
Representative calcium imaging traces of SGCs and neurons following application of glutamate agonists and antagonists. Following direct application of A: AMPA (50 µM), B: NMDA (100 µM), C: DHPG (100 µM) and D: KA (30 µM) both neurons and SGCs responded to all three agonists. Following a HEPES buffer wash the same cells were then given the appropriate selective antagonist: A^1^, E^1^: CNQX (100 µM); B^1^, F^1^: APV (100 µM); C^1^, G^1^: AP3 (1 mM) and D^1^, H^1^: UBP310 (0.5 µM) for 5 minutes prior to second application of agonist. In the presence of its antagonist, the effect of each agonist was blocked (A^1^–D^1^ SGCs; E^1^–H^1^ neurons). A^2^–H^2^, ten minutes after HEPES buffer wash, KCl (50 mM) was given to identify neurons in the recorded field and ionomycin (20 µM) was added to test cell viability at the end of the experiment. Lines show the duration of application. Scale bar = 5 minutes.

**Figure 5:**
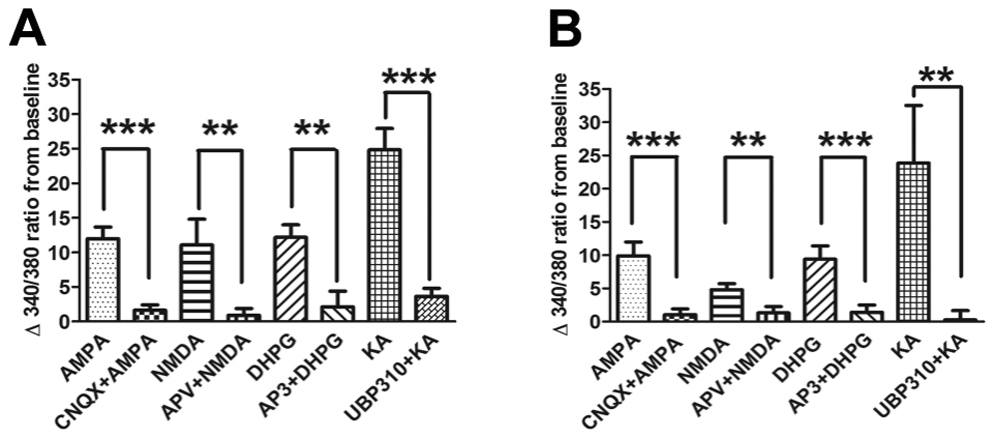
Summary data of calcium imaging experiment on glutamate receptor activation. Effects of each agonist were significantly blocked in the presence of their respective antagonists (A, SGCs; B, neurons) in calcium imaging experiments. Pre-treatment of the selective appropriate antagonist, resulted in a reduction in activation by the agonist of both neurons and SGCs. Cell numbers used in this summary bar graph (neuron/SGC): AMPA (25/35), NMDA (12/9), DHPG (20/5) and KA (11/22). Each agonist used 3–4 cultures from 2–3 animals. Each column represents the mean ± S.E.M. ***, P<0.001; **P<0.01.

We examined which subtypes of glutamate receptors were responsible for the increase in cytosolic Ca^2+^. Ionotropic and group I metabotropic receptor agonists AMPA, NMDA, KA, and DHPG were used to stimulate cells. The selective antagonists CNQX, APV, AP3 and UBP310 were used to block the effect of each agonist (Figures 4 and 5). As shown in Figure 4, all four agonists caused an immediate Ca^2+^ influx, which lasted through the entire recording period (10 minutes) in both SGCs (Figure 4 A–D) and neurons (Figure 4 E–H). To confirm the selectivity of the agonists, 5 minutes incubation with the appropriate antagonist was able to block the effect of a second application of agonists in SGCs (Figure 4 A^1^–D^1^) and neurons (Figure 4 E^1^–H^1^). The summary data are shown in both Figure 5 and Table 2. Similar results were found in both neuron and SGC populations.

**Table 2:** Neurons and SGCs responding to glutamate or selective agonists with or without specific antagonists using calcium imaging.

Agonist vs. Antagonist+Agonist	Responding Neurons#/total N (%)	Δ 340/380 ratio neurons	Responding SGCs#/total N (%)	Δ 340/380 ratio SGCs
Glutamate	51/56 (91)	12.7±1.8***	34/40 (85)	7.81±1***
AMPA vs. CNQX+AMPA	25/29 (86)	9.8±2.1 vs. 1.0±0.8***	35/37 (95)	11.9±1.7 vs. 1.6±0.8***
NMDA vs. APV+NMDA	12/23 (52)	4.8±0.9 vs. 1.3±0.9**	7/14 (50)	11.1±3.7 vs. 0.9±0.9**
KA vs. UBP310+ KA	11/13 (85)	23.8±8.6 vs. 0.3±1.4**	22/24 (92)	24.8±3.1 vs. 3.6±1.2**
DHPG vs. AP3+ DHPG	20/21 (95)	9.4±2.0 vs. 1.4±1.1***	5/6 (83)	12.2±1.8 vs. 2.1±2.3**

Mean ± S.E.M.

** P<0.01;

*** P<0.001.

To confirm that the reduction in Ca^2+^ influx following antagonist application was not the result of receptor sensitization [38], we tested whether glutamate and selective glutamate agonists can induce Ca^2+^ influx after repeated application with washes between applications. We found that all agonists were capable of increasing the 340/380 ratio after washing 2 to 3 times (data not shown). From this we conclude, the reducing Ca^2+^ influx ratio after pretreatment of antagonist was not due to desensitizing of the receptors.

### Activation of DRG Neuronal Perikaryal Glutamate Receptors causes Inward Currents

**Figure 6:**
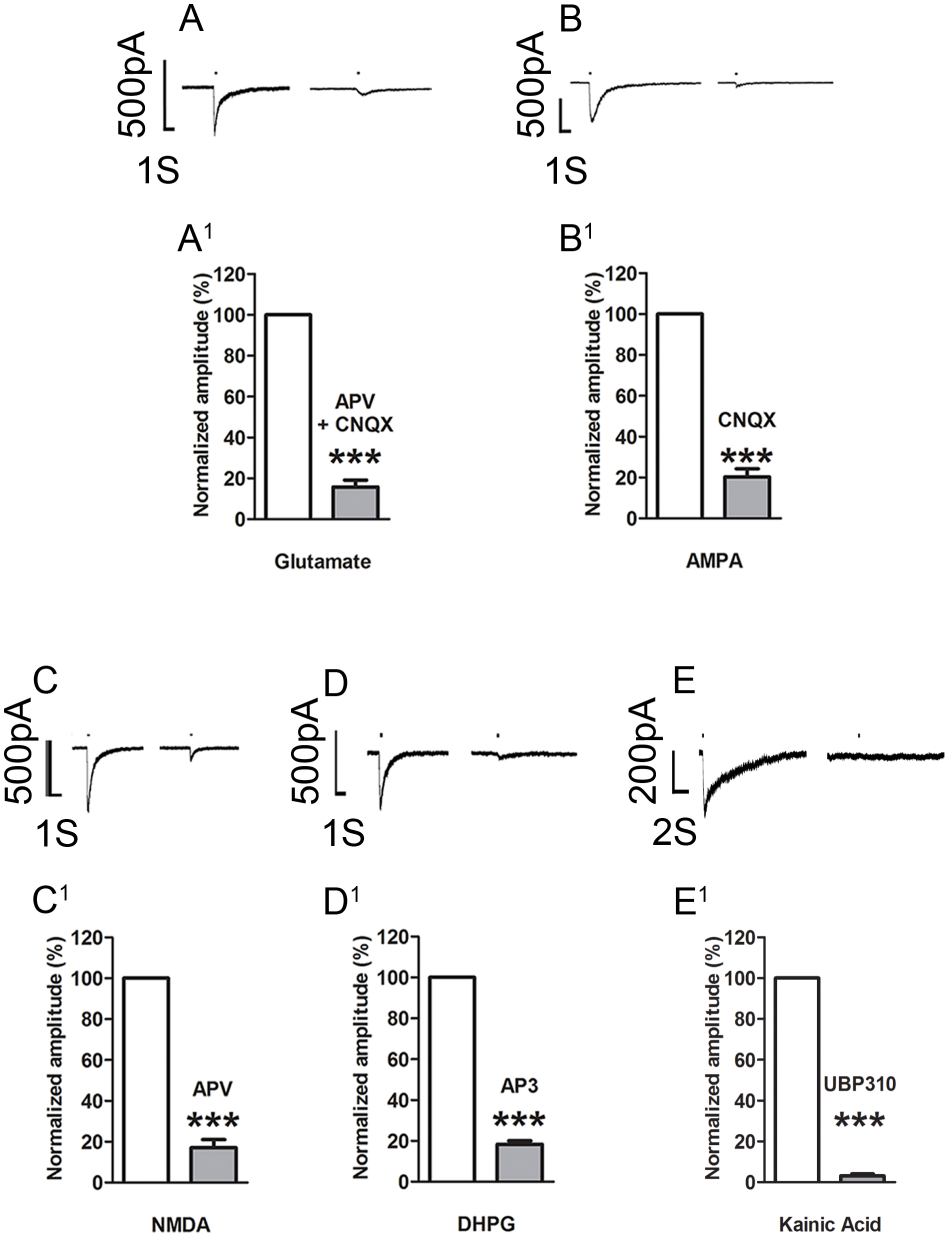
Whole cell patch recordings of small neurons from *ex-vivo* dorsal root ganglia. A–E, puff application of glutamate or the receptor selective agonists induced inward currents which were blocked (right trace) by bath application of the appropriate selective antagonist(s). A^1^–E^1^, bar graphs showing the reduction in standardized amplitude of the receptor induced currents in presence of the appropriate antagonist. See Table 1 for drug concentrations. The bars above all the traces indicate agonist application time (200 ms). The decay time for AMPA induced current is: 520.0±70.4 ms; NMDA: 461.9±39.7 ms; Glutamate: 537.0±50.8 ms; KA: 512.4±482.4 ms; DHPG: 90.1±25.8 ms. All data are expressed as mean ± SEM. ***P<0.001.

Patch clamp recordings were made from 122 neurons of which 45 (36.9%) showed responses with inward currents to application of 1 mM glutamate. Pooled data from these neurons show the amplitude of the current was 234.3±39.9 pA (Table 3). The currents induced by glutamate were blocked by bath application of 50 µM APV and 10 µM CNQX (Figure 6A 84.2% ±3.4 amplitude decrease from agonist alone, N = 8, P<0.001) indicating that the glutamate response was mediated at least by AMPA and NMDA receptors. To further investigate the receptors underlying the glutamate response, neurons were tested with selective agonists and antagonists (Figure 6; Table 3). In the presence of the ionotropic antagonists, CNQX, APV, AP3 or UBP310 currents induced by glutamate, ionotropic agonists AMPA, NMDA, KA or metabotropic agonist DHPG, respectively, were significantly reduced by ∼80% (Table 4). These results showed that ionotropic (NMDA, AMPA, KA) and metabotropic (mGluR1/5) receptors were functionally expressed on the perikarya of DRG neurons.

**Table 3:** Neurons responding to glutamate or selective receptor agonists in *ex-vivo* patch-clamp experiments.

Agonist	Total (N)	Cell responding (%)	Mean of inward current (pA)
Glutamate	122	37	234.3±39.9
AMPA	38	29	275.8±45.1
NMDA	55	33	554.0±94.8
KA	21	81	128.2±32.0
DHPG	50	24	89.9±38.2

Mean ± S.E.M.

**Table 4:** Neurons responding to selective antagonist following agonist treatment.

Antagonist(Figures 6A ^1^–6E^1^)	Total (N)	Cell responding (%)	Mean of inward currents induced by second application of agonist in the presence of antagonist (pA)	Mean of inward currents reduction from first application of agonist (%)
APV+CNQX	8	100	37.0±6.2	84.2±3.4***
CNQX	7	100	34.4±8.5	79.7±4.0***
APV	7	100	89.0±15.1	83.0±3.9***
UBP310	3	100	5.1±1.3	96.9±1.0***
AP3	7	100	15.1±3.2	81.7±1.7***

Mean ± S.E.M.

*** P<0.001.

### Glutamate Immunosignal is Increased after CCI of the Sciatic Nerve

**Figure 7:**
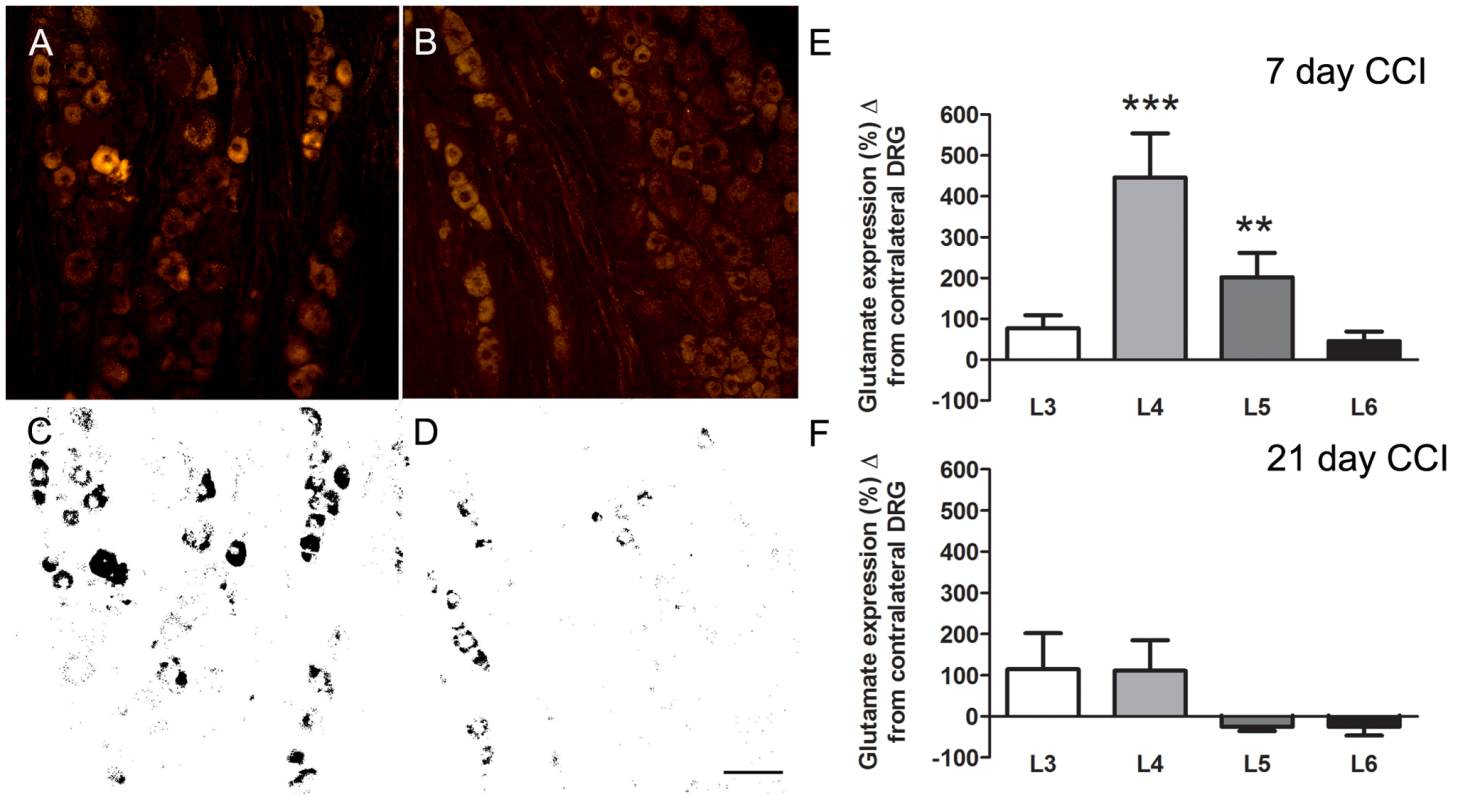
Representative images from an L4 DRG immunostained for glutamate following CCI of the sciatic nerve. Increased glutamate immunolabeling is seen in the DRG ipsilateral to the sciatic CCI (A standard, C thresholded image) compared to the contralateral side (B standard, and D thresholded image). E, F shows the % difference in glutamate immuno-expression between the ipsi and contralateral L3 - L6 DRGs at 7 (E) and 21 (F) day post-CCI-SN animals. Scale bar = 50 µm. Data expressed as mean ± SEM. **, P<0.01; ***P<0.001 ipsilateral vs. contralateral.

Seven days following CCI of the sciatic nerve, glutamate immunosignal was measured in neurons of spinal ganglia at the L3 to L6 levels ipsilateral to the CCI and compared to the contralateral side (Figure 7A–E). The L4 and L5 ganglia that provides the majority of axons to the sciatic nerve showed increases of 446% (±108%) and 202% (±60%,) while glutamate levels in the L3 and L6 were not significantly different from the non-ligated side (L3, 77% ±31%; L6, 45% ±23%). Twenty-one days after CCI, glutamate expression in all ganglia (L3–L6) was not significantly different compared to the contralateral side (Figure 7 F). Comparison of glutamate immuno-expression on the contralateral side between days 7 and 21 showed no significant difference (P>0.05) indicating that the reduction in the ipsilateral expression at 21 days following ligation was not due to an increased expression of the contralateral side. Animals were also monitored behaviorally every other day using von Frey hairs. All animals exhibited a decreased pain threshold on the side of the CCI of the sciatic nerve (left side), which was significant at 7 and 21 days (Day 0, left = 72.44 g, ±3.99, right 71.63 g, ±5.19 g, p = ns; Day 7, left = 31.08 g, ±2.92 g right 70.84 g, ±5.03 g, p<0.001; Day 21, left = 45.85 g, ±4.59 g, right 86.52 g, ±1.08 g p = <0.001).

### Glutamate Expression is Increased in Trigeminal Ganglion after CCI of the ION

**Figure 8:**
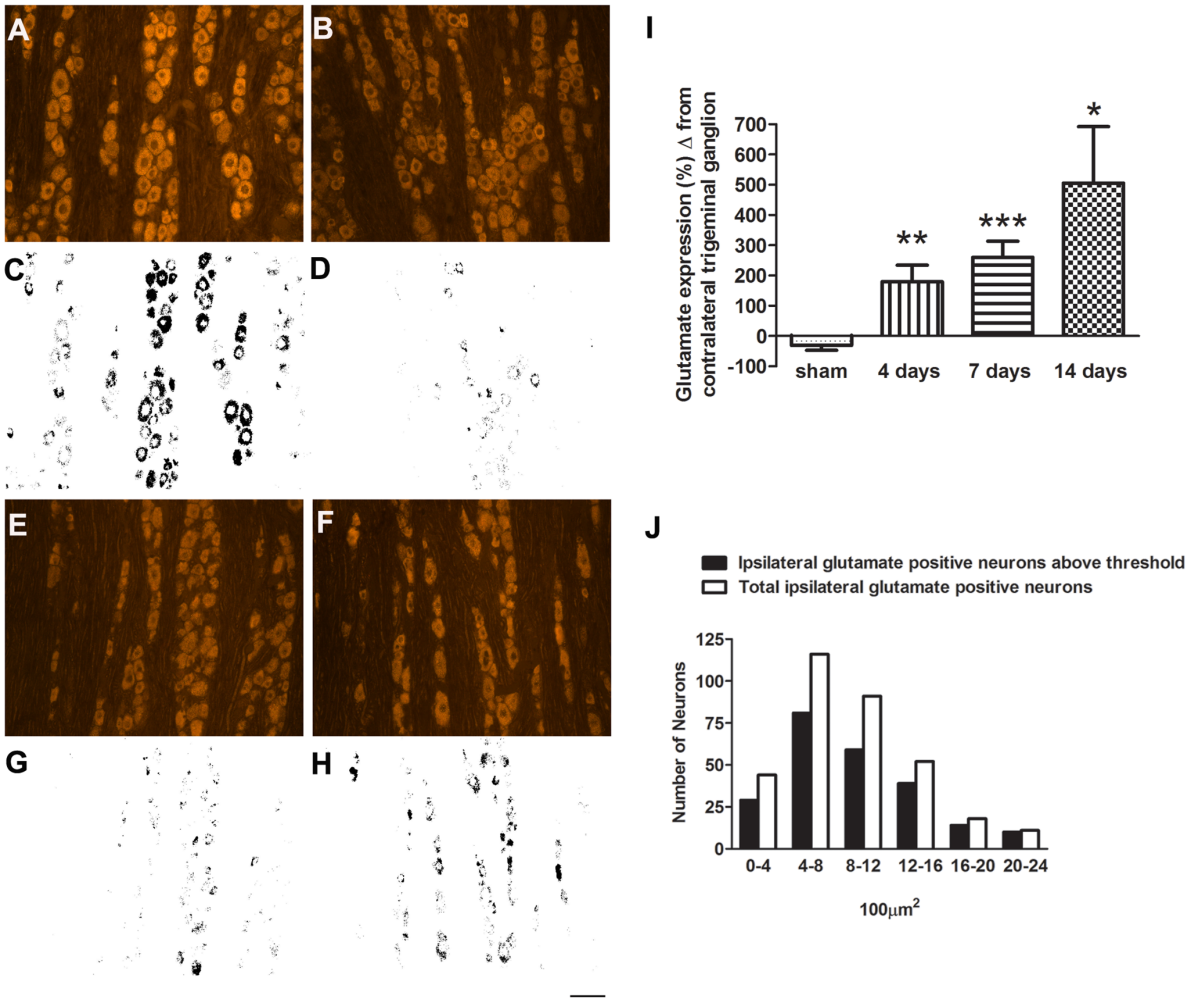
Representative images from a trigeminal ganglion immunostained for glutamate following CCI of the ION. Increased glutamate immunolabeling is seen in the trigeminal ganglion ipsilateral to the CCI (A, standard, C, thresholded image) compared to the contralateral side (B, standard, D, thresholded image). I, shows the % difference in glutamate immuno-expression between the ipsi and contralateral trigeminal ganglion for sham, 4, 7 and 14 days post CCI. Ganglia from sham-operated rats (E, standard, G, thresholded image) showed no difference compared to contralateral ganglia (F, standard, H, thresholded image). J, white bars show total glutamate immunopositive cells sorted by size from 9 sections of three 14-day CCI-ION trigeminal ganglion and the black bars show the number glutamate positive cells above threshold. Scale bar = 50 µm *, P<0.05; **, P<0.01; ***, P<0.001.

**Figure 9:**
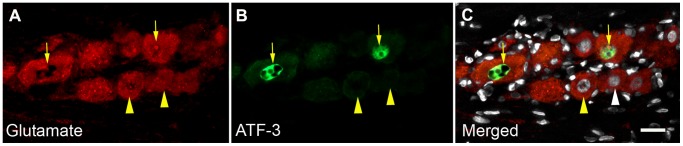
Relation between injured neurons and change in glutamate expression. Seven days following CCI of the inferior orbital nerve many neurons show increased levels of glutamate immunosignal (A, arrows and see Figure 8). Not all these cells are double labeled for ATF3 (B, arrows) a marker of neuronal injury. The neurons indicated with arrowheads are also above threshold but not ATF3 immuno positive. This is not due to the nucleus being out of the plane of section as DAPI staining (C, arrowheads) shows nuclei are present. Scale bar = 30 µm.

To determine whether an increase in somatic glutamate also occurred in the trigeminal ganglia, we performed glutamate immunocytochemistry at days 4, 7 and 14 post-CCI of the ION (Figure 8). Rats were also tested behaviorally every other day using von Frey hairs. Decreased pain thresholds were observed on the side of the nerve injury (left side) throughout the study period (Pain score. Day 0, left = 2.0±0.5, right = 2.0±0.5, p = ns; Day 7, left = 3.5, ±0.5, right = 2.1±0.2, p = 0.42; Day 14, left = 3.8±0.4, right = 2.3± = 0.3, p = 0.002). Increased glutamate expression was observed in the ipsilateral compared to the contralateral ganglia at all three time points post CCI (Figure 8 I, 4 days, 180±55%; 7 days, 260±53%; 14 days, 506±186%). In animals with sham surgery there was no difference in glutamate expression between left and right trigeminal ganglia (Figure 8 I). The neurons showing increased glutamate expression were not confined to a specific cell size but occurred through the entire population (Figure 8 J). To show the increased in glutamate occurred in the territory of the injured ION we double labeled for both glutamate and ATF3 (Figure 9). ATF3 is a marker of neuronal injury [39]. While the increase in glutamate was in the portion of the trigeminal ganglia that harbors the somas of neurons with projections in the ION, not all neurons with increased glutamate expression had ATF3 immunopositive nuclei (Figure 9).

## Discussion

### Glutamate is Released into the Extracellular Space within the Sensory Ganglion

It is well established that glutamate is released from both the central and peripheral axonal endings of primary sensory neurons [11], [12], [40] but the possibility that glutamate is also released from the cell body (soma), although not a new concept, is not widely recognized. More than two decades ago, Larson’s group reported that cultured whole ganglia released glutamate in response to KCl or capsaicin [26] stimulation. Our results, and those of others [24], [25], confirm that glutamate is released from dissociated DRGs and trigeminal ganglia following KCl stimulation. When cortical [41] or DRG primary cultures (present study) were pretreated with TBOA, an inhibitor of glutamate uptake with a higher affinity for glial transporters, the amount of extracellular glutamate following KCl treatment increased markedly. This is evidence for the key role played by SGCs in regulating glutamatergic transmission within the ganglion [17] given that most of glutamate reuptake occurs through glial transporters [21], [42], [43]. This observation complements a previous *in-vivo* study in the trigeminal ganglion when we silenced the expression of GLAST, one of the two glial glutamate transporters. For the duration of the silencing (3–4 days) all rats presented a neuropathic-like pain behavior in the innervation territory of the trigeminal nerve [22]. We postulated that the behavioral changes were due to increased extracellular glutamate resulting from reduced glial uptake, a situation mimicked by the addition of TBOA to mixed cultures in the present study.

Although there are no synapses in the ganglion, or at the peripheral terminals where glutamate is known to be released [44], a vesicular mode of release is not excluded [44], [45]. Some components of the vesicular docking mechanism are seen at the level of the somatic membrane within the ganglion (Figure S1). Alternatively, channels and transporters through which non-vesicular release of glutamate occurs are also present, including the cystine-glutamate antiporter [46], gap junctions (connexin 43) [23], the P2X7 channel [28], [47], or reverse transport through GLAST or GLT1 [21], [43]. Although we did not see glutamate immuno-staining in SGCs it is possible that this due to the limited sensitivity of immunocytochemistry. Studies showing that astrocytes are able to synthesize and release glutamate [48], [49], [50] suggest that this might also occur for SGCs, yet it remains to be shown.

### Glutamate Receptors in Primary Sensory Neurons

The weight of evidence supports the idea that glutamate is released from neuronal cell bodies within sensory ganglia but this leaves open the question of whether such release has any functional consequences. In other words, are there functional glutamate receptors on the perikarya within the ganglia? Anatomically, NMDA, AMPA, kainate and metabotropic receptors have been reported in primary sensory neurons [13], [14], [15]. NMDA receptors are expressed in 40 to 60% of primary sensory neurons most of which are nociceptive (i.e. small diameter) while AMPA is expressed in about 34% of primary sensory neurons [15], [16]. Kainate receptors are the only glutamate receptor found exclusively in small diameter primary sensory neurons [13] suggesting a closer association between this receptor and nociception in the periphery. Metabotropic GluR 1/5 receptor are also expressed mainly by small diameter neurons (30 microns) but their distribution is limited (less than 7% of DRG neurons) [14].

The immunocytochemical identification of receptor proteins in the soma does not necessarily indicate these are functional receptors as they could represent subunits of glutamate receptors that are destined to be exported to the central or peripheral axon terminals where they are inserted into the membrane. Evidence for the presence of functional receptors comes from the present study and previous pharmacological and electrophysiological studies showing that application of glutamate receptor agonists depolarize cultured DRG neurons and activate Ca^2+^-dependent currents. There are some discrepancies in the results from previous studies when it comes to which receptor is involved at the soma. Lovinger and Weight concluded that NMDA but not kainate receptors were active on the perikarya of a large proportion primary sensory neurons [51] while Huettner, using whole-cell voltage clamp, found that most neurons exhibit strong responses to kainate receptor agonists, but less response to AMPA and NMDA agonists [52]. In contrast, Agrawal and Evans [53] recorded strong depolarization to KA and to a lesser degree to AMPA in dorsal root fibers, but found little or no response when KA or other excitatory amino-acids were applied directly to the ganglia. For metabotropic receptors, it has been shown that the mGluR5 (but not mGluR1) receptor subtype underlies the glutamate-mediated oscillatory increases in Ca^2+^ at the soma [54], [55] while group II/III mGluRs appear to have an inhibitory effect [56].

Our data expands previous studies by showing that all three types ionotropic receptors as well as group 1/5 mGluR are present on the perikarya of primary sensory neurons and all respond to the appropriate selective agonists with inward currents. Of particular note is that our results were obtained using two different methods on different tissue preparations, Ca^2+^ imaging studies on dissociated ganglia and patch clamp studies on intact, *ex-vivo*, whole ganglia. In the latter case because the neuron/SGC relationship is preserved and the neurons retain their central and peripheral axons, it is unlikely that the glutamate receptor response can be artifactually induced by the dissociation or culture conditions.

Our Ca^2+^ imaging studies have provided additional data not available from the *ex-vivo* experiments. Calcium imaging shows that AMPA [57], NMDA, kainate, and mGlu [14] receptors on SGCs (Figure S2), respond to the appropriate selective agonists, meaning that they are functional. The mGluR response confirms an earlier observation in the nodose ganglion by Shoji and colleagues [58] who showed an increase in intracellular Ca^2+^ in both neurons and surrounding SGCs in response to glutamate or the mGluR agonist (t-ACPD). This latter result indicates that SGCs in autonomic ganglia also express functional glutamate receptors.

The kinetics of Ca^++^ influx were similar when either AMPA, NMDA or DHPG was applied although the kinetics of activation would be expected to be different for the different receptors. The similarity in responses in our experiments can be explained by the fact we are sampling at 300 milliseconds/frame, so we are unable to differentiate any event faster than 300 milliseconds NMDA and AMPA channels open in the 1–10 milliseconds range while metabotropic receptors activate in the 50–100 milliseconds range and thus are outside our temporal resolution. We also do not know the origin of AMPA-mediated calcium entry; Fura 2A (calcium indicator dye) only indicates the amount of calcium change inside the cell. The calcium responses we observed could result from the calcium permeable AMPA or NMDA receptors and/or activation of voltage gated calcium channels all of which open and close within millisecond range and cannot be differentiated with our calcium imaging protocol. Finally it should be noted that the application method is such that the drugs takes several seconds to dissipate, so the recording is from a large number of receptors being activated over several seconds and therefore it is not possible to determine individual channel kinetics.

The importance of functional glutamate receptors on primary sensory cell bodies is fairly straightforward. It means that extracellular glutamate in the ganglia can change the membrane potential of the ganglion neurons. The consequences in terms of changes in sensory processing will depend on the magnitude of the membrane threshold changes, whether ionotropic or metabotropic channels are involved and differences between physiological classes of neurons. This adds to the growing recognition of complex chemical messenger interactions between neurons and SGCs within sensory ganglia. The most established is through the release of ATP that activates local purinergic receptors [29], [59], [60], [61]. In response to stimulation, SGCs have been shown to release a variety of cytokines [29], [60], [62].

### Glutamate Increases in the Cell Body of Primary Sensory Neurons after Peripheral Nerve Injury

The number of glutamatergic neurons in primary sensory ganglia has been previously reported as being between 30 and 70% [6], [7], [44], [63], [64]. Quantitation using immunocytochemistry has to be treated with caution as technical issue such as antibody sensitivity can bias the results and in fact several of the above authors have noted variable results depending on the method used [64] or use of colchicine [7]. The latter study [7] observed a large increase in the number of glutamate positive perikarya after colchicine treatment which suggests that negative results might result from insensitivity of some immunocytochemical methods. It can be seen in our Figures 7 and 8 that all neurons in the trigeminal ganglia and DRG appear to have some amount of staining and determining what is background is somewhat arbitrary. However, it should be noted that glutamate has non-neurotransmitter functions in cell metabolism [65], [66] therefore low levels of immunoreactivity need not necessarily be artefactual. Because the number of immunopositive cells counted depends on where the cut-off for background staining is set we did not try and estimate the total number of glutamate immunopositive neurons but rather we determined the increase in glutamate expression following injury. We also used a relatively high threshold that was the same for both the injured and non-injured ganglia (they were processed side-by-side on the same slide) and reported the change in immunolabeling of the injured side compared to the uninjured side.

A significant increase in glutamate immuno-staining was seen, as early as 4 days post-CCI in the trigeminal ganglion and at 7 days in the L4 and L5 DRGs. This increase lasted until day 14 post-CCI for both the trigeminal and lumbar sensory ganglia. The rise in glutamate occurred in neurons that were injured by the CCI or were located in the region of the ganglion containing injured neurons. This is demonstrated both by the double label studies with glutamate and ATF3 and also by the finding that the increase in glutamate was localized to the V2 division of the trigeminal ganglion where axons in the ION arise and throughout the L4 and L5 spinal DRGs ipsilateral to the sciatic nerve ligation. Approximately half the neurons of L4/L5 DRGs project axon into the sciatic nerve [67] in contrast to only 0.4% the L6 and 1.2% of the L3 DRG neurons [68]. Accordingly, no significant increase in glutamate was seen in the L3 and L6 ganglia. The increase in glutamate was seen in neurons of all diameters and is consistent with the fact that all sizes of axon are injured in the CCI model [69].

While there are no previous reports of glutamate increasing in the sensory ganglion after peripheral nerve injury, Westlund et al [70] reported an increase in the number of glutamate immunopositive in peripheral fibers from 25% to over 60% following induction of arthritis; the ganglia was not examined. The glutamate increase we report in injured neurons as indicated by ATF3 [39] expression may be due to increased activity or from decreased axonal transport. Why glutamate increases in non-injured (i.e. ATF3 immunonegative) neurons is not clear. It could be due to a paracrine effect via factors such as neurosteroids [71], chemokines [72], serotonin [73], or glial cell line-derived neurotrophic factor [59] released in the ganglion by injured neurons. The increase in glutamate is likely to be associated with increased release given that after nerve lesion the vesicular transporter VGLUT2 also increases in small diameter ganglion neurons [74], voltage activated Ca^2+^ channels are upregulated, Ca^2+^ dependent of glutamate release increases, and reuptake decreases [75], [76], [77], [78], [79]. It is unclear what proportion of the glutamate seen in the soma of primary sensory neurons (baseline or after nerve injury) is released in the ganglion and what proportion from peripheral or central terminals.

While the importance of glutamate as a neurotransmitter in the CNS and in primary afferent terminals is well documented [80], [81], [82], the role of glutamate within the ganglion is not established. The present data showing that CCI is accompanied by an increase in glutamate in the soma of sensory ganglion neurons suggesting that glutamate in the ganglion may be the key in the initial expression of neuropathic pain and triggering the events in the CNS that leads to chronic pain.

Even with the evidence that glutamate can act locally in the ganglion to activate receptors, there is the question of whether intraganglionic glutamate activity has any relevant behavioral effects. We have previously shown that knockdown of components of the glutamate uptake and recycling mechanism in SGCs results in quantifiable spontaneous pain behavior, ipsilateral allodynia and ipsilateral hyperalgesia [22], [23]. We hypothesized that interrupting glutamate uptake and recycling by SGCs resulted in an increase in extracellular glutamate and activation of glutamate receptors, which may in turn change the excitability of the ganglion neurons. More experiments will have to be done to address this question. This model is analogous to pharmacologically blocking glutamate uptake in the spinal cord, which results in nociceptive responses [83], [84].

If the nociceptive actions of glutamate release within the ganglion is confirmed, then altering glutamatergic transmission within the ganglion might be an avenue for pain therapy. The rationale of targeting glutamate in the early stages of nerve injury is supported by data showing that glutamate uptake is less efficient after nerve injury [85], [86], [87] and restoring the expression of the GLT-1 in the spinal cord decreases neuropathic pain behavior [78]. While one could argue that decreasing glutamate in the ganglion might offset some of the inhibitory effect glutamate has through its group II and III metabotropic receptors, recent evidence suggest that at least the mGluR7 receptor is down-regulated after injury, which contributes to increased nociception [77]. Finally, the concept that peripheral glutamatergic transmission could be a significant contributor to changes in gene expression in the sensory neurons is supported by the finding that selective knockdown of the NMDA receptor in primary afferent neurons prevents the sensitization of primary afferents [16].

### Conclusion

This study provides evidence that all the components for receptor mediated glutamatergic transmission are present in the sensory ganglia and that glutamate expression increases in primary sensory neurons following nerve injury. These finding further support the idea that peripheral, and intraganglionic in particular, glutamatergic transmission can be a factor in the initiation and/or maintenance of pain following nerve injury.

## Supporting Information

Figure S1 Immunocytochemistry of some proteins associated with glutamate vesicle-packaging and release. Each row of images (A–D) show the vesicle associated protein, SGCs labeled with glutamine synthetase and the merged image. Some of these proteins are found only in neurons (SNAP25, A), some only in SGCs (Cellubrevin, C) and other occur in both cell types (synaptobrevin, B and secretory carrier-associated membrane protein 1 [SCAMP1], D). Scale bar: A, C, D = 10 µm, B = 30 µm.
(TIF)

Figure S2 Immunocytochemistry of glutamate receptor expression in sensory ganglia. Some of the glutamate receptors are found in both neurons and SGCs (NR2A, A; GluA4, C3; mGluR8, D3). Subunit GluK2 of kainate receptors is found only in SGCs (B). Double label of glutamine synthetase (GS) a marker for SGCs and GluA4 or mGluR8 are shown from C1to C3 and D1 to D3 respectively. Arrows: SGCs, Arrow head: SGC nucleus. Scale bar: A = 30 µm, B = 25 µm, C, D = 30 µm.
(TIF)
