# Supplementary figures and images for: Evidence for Glutamate as a Neuroglial Transmitter within Sensory Ganglia
Source: PLoS One. 2013 Jul 2;8(7):e68312. doi: 10.1371/journal.pone.0068312 (PMC3699553; doi:10.1371/journal.pone.0068312)

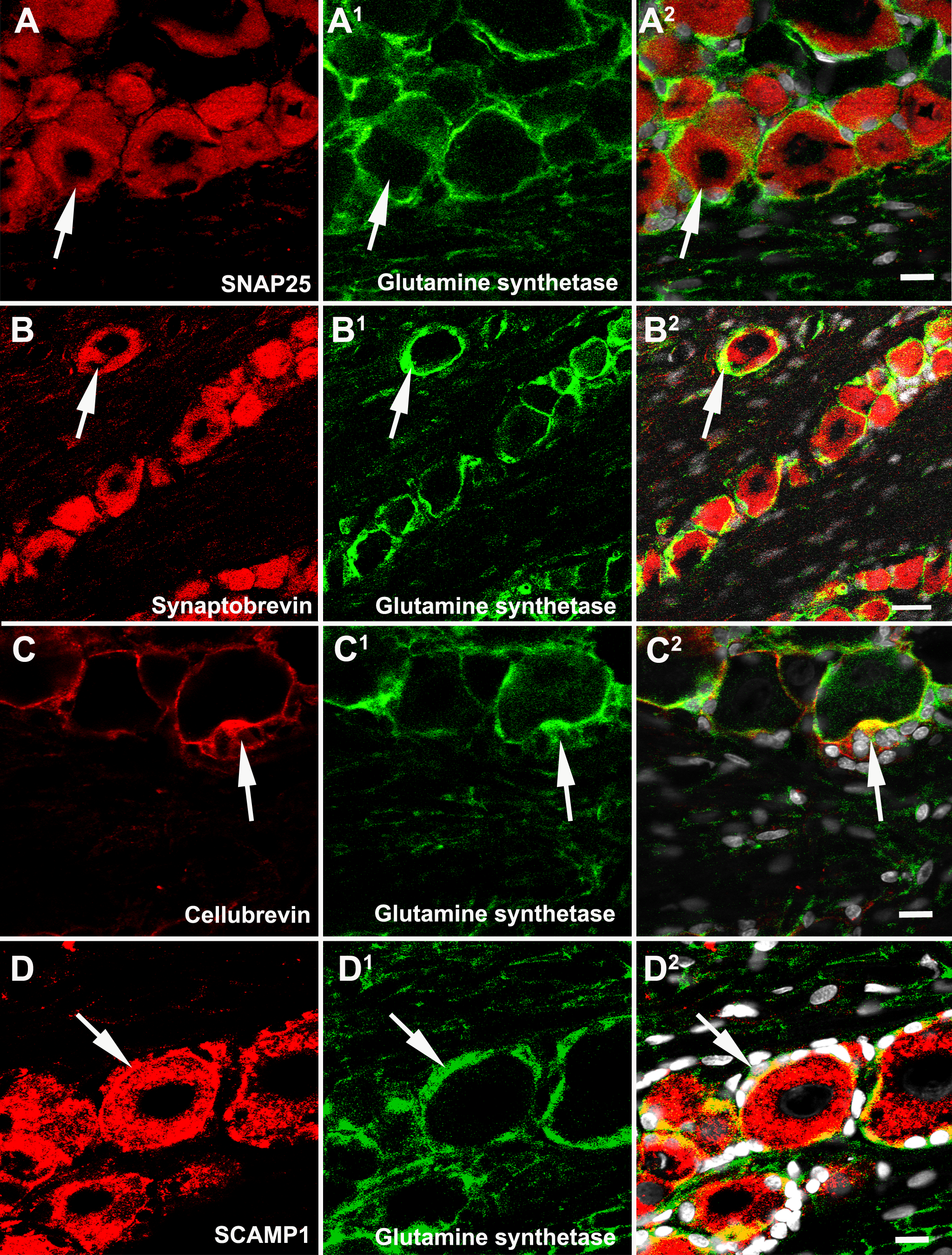

Supplement: Figure S1 — Immunocytochemistry of some proteins associated with glutamate vesicle-packaging and release. Each row of images (A–D) show the vesicle associated protein, SGCs labeled with glutamine synthetase and the merged image. Some of these proteins are found only in neurons (SNAP25, A), some only in SGCs (Cellubrevin, C) and other occur in both cell types (synaptobrevin, B and secretory carrier-associated membrane protein 1 [SCAMP1], D). Scale bar: A, C, D = 10 µm, B = 30 µm. (TIF) [file pone.0068312.s001.tif]

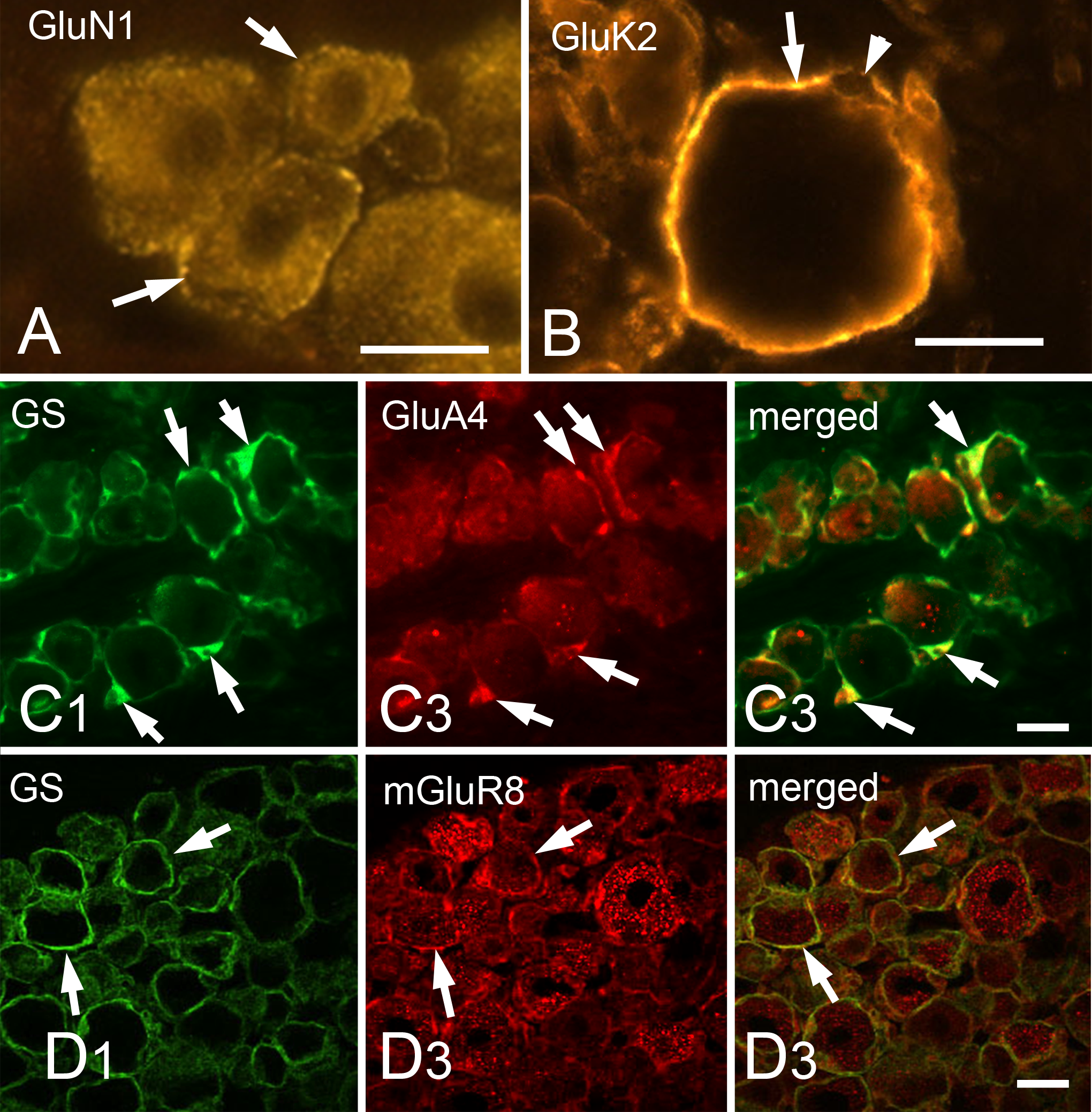

Supplement: Figure S2 — Immunocytochemistry of glutamate receptor expression in sensory ganglia. Some of the glutamate receptors are found in both neurons and SGCs (NR2A, A; GluA4, C3; mGluR8, D3). Subunit GluK2 of kainate receptors is found only in SGCs (B). Double label of glutamine synthetase (GS) a marker for SGCs and GluA4 or mGluR8 are shown from C1to C3 and D1 to D3 respectively. Arrows: SGCs, Arrow head: SGC nucleus. Scale bar: A = 30 µm, B = 25 µm, C, D = 30 µm. (TIF) [file pone.0068312.s002.tif]
